# The Role of Curcuminoid in Preventing and Repairing Damage to the Organ of Corti in *Rattus norvegicus* Diabetes Mellitus Model as Assessed by DPOAE Examination with Plasma Levels of HIF-1α and VEGF-A

**DOI:** 10.1055/s-0045-1811641

**Published:** 2026-03-19

**Authors:** Muhammad Edy Syahputra Nasution, Tengku Siti Hajar Haryuna, Juliandi Harahap, Syafruddin Ilyas, Dharma Lindarto

**Affiliations:** 1 Doctoral Program in Medical Science, Faculty of Medicine, Universitas Sumatera Utara, Medan, Sumatera Utara, Indonesia; 2 Department of Otorhinolaryngology–Head and Neck Surgery, Faculty of Medicine, Universitas Muhammadiyah Sumatera Utara, Medan, Sumatera Utara, Indonesia; 3 Department of Otorhinolaryngology–Head and Neck Surgery, Faculty of Medicine, Universitas Sumatera Utara, Medan, Sumatera Utara, Indonesia; 4 Community Medicine Department, Faculty of Medicine, Universitas Sumatera Utara, Medan, Sumatera Utara, Indonesia; 5 Study Program of Biology, Faculty of Mathematics and Natural Sciences, Universitas Sumatera Utara, Medan, Sumatera Utara, Indonesia; 6 Department of Internal Medicine, Faculty of Medicine, Universitas Sumatera Utara, Medan, Sumatera Utara, Indonesia

**Keywords:** curcuminoid, HIF-1α, otoacoustic emission, SNR, VEGF-A

## Abstract

**Introduction:**

Cells in diabetes mellitus (DM) respond to low oxygen by increasing hypoxia-inducible factor-1 (HIF-1), which impacts vascular endothelial growth factor (VEGF). Although curcuminoids show therapeutic promise, no validated options are available to repair organ of Corti damage caused by DM.

**Objective:**

The present study aims to evaluate the effects of curcuminoid administration on the signal-to-noise ratio (SNR) and plasma levels of HIF-1α and VEGF-A in a DM model using *Rattus norvegicus* .

**Methods:**

An in vivo experimental design was employed with a double-blind control set up. Twenty-five male Wistar rats were divided into 5 groups: group 1 (DM without curcuminoids), groups 2 and 3 (DM with curcuminoids at 200 and 400 mg/kg body weight for 10 days), and groups 4 and 5 (DM with curcuminoids at 200 and 400 mg/kg body weight for 13 days). Diabetes mellitus was induced through an intraperitoneal injection of alloxan (150 mg/kg body weight). Distortion product otoacoustic emission (DPOAE) tests were performed. Enzyme-linked immunosorbent assay (ELISA) was used to measure plasma levels of HIF-1α and VEGF-A.

**Results:**

Significant differences were observed in SNR values, HIF-1α, and VEGF-A ( *p* < 0.05). A significant correlation was found between SNR and HIF-1α (r = - 0.553; *p* = 0.004) and VEGF-A (r = - 0.564; *p* = 0.003), indicating that lower levels of these factors were associated with higher SNR.

**Conclusion:**

Curcuminoids can prevent and treat outer hair cell damage in the organ of Corti due to DM, as shown by improvements in plasma HIF-1α, VEGF-A levels, and SNR values.

## Introduction

The International Diabetes Federation estimates that the number of adults with diabetes will reach 784 million by 2045. 1 Diabetes mellitus (DM) is associated with several macrovascular and microvascular complications, including increase in thickness of the basal membrane of the capillaries in the stria vascularis of the cochlear lateral wall, which can lead to hearing impairment. 2 A meta-analysis found that the incidence of hearing impairment is elevated in subjects with type 1 and type 2 diabetes compared with the general population without diabetes, with a combined odds ratio (OR) of 2.15 (95%CI: 1.72–2.68). 3 4

A well-established relationship exists between DM and hearing impairment. Distortion product otoacoustic emission (DPOAE) testing has revealed subclinical changes in cochlear function among patients with DM. 5 Similarly, otoacoustic emissions (OAE), which reflect the condition of inner ear hair cells, have been found to be significantly lower in diabetic patients. 6 Hearing loss and cochlear histopathological changes occur due to thickening of blood vessel walls in the modiolus and capillary narrowing in the stria vascularis, leading to reduced blood flow to the inner ear. 7 This vascular compromise results in the loss of hair cells within the cochlear organ of Corti. 8 The outer hair cells of the organ of Corti are particularly vulnerable to damage in DM because they lack the ability to regenerate spontaneously after injury. 6 9 Hair cells in the organ of Corti serve as auditory sensors, converting mechanical energy into electrochemical signals. 6 The outer hair cells specifically function to amplify sound and regulate the cochlea's sensitivity to different frequencies. 10 Macrovascular and microvascular dysfunction, which leads to reduced blood flow, altered oxygen exchange, and impaired ion transport, is a direct complication of DM that significantly impacts cochlear hair cell. 11 Histological studies in diabetic animal models have confirmed outer hair cell loss, thickening of blood vessel walls in the modiolus and stria vascularis, and reduced cochlear blood flow. 12

Upregulated hypoxia inducible factor- α (HIF-1α) in DM has been identified as a contributing factor to cochlear damage, leading to hearing impairment. In DM, chronic hypoxia induced by metabolic disturbances can trigger increased expression of HIF-1α, which in turn activates inflammatory responses and oxidative stress within the cochlea. 13 14 One of the key consequences of HIF-1α upregulation is the dysregulation of vascular endothelial growth factor A (VEGF-A), which plays a crucial role in angiogenesis and in the maintenance of vascular function in the cochlea. 15 In DM, VEGF-A dysregulation can lead to increased vascular permeability, reduced blood flow, and impaired nutrient and oxygen delivery to the cochlea, potentially exacerbating hearing impairment in diabetic patients. 16 17 Targeting HIF-1α and VEGF-A may serve as a promising therapeutic approach to mitigate vascular damage, preserve cochlear function, and prevent or slow the progression of diabetes-related hearing impairment.

In the present study, we used DPOAE because it is an objective, noninvasive, and more sensitive method for assessing early or subclinical cochlear damage. 18 Rats are commonly used and valuable for hearing research. 19 Additionally, diabetic rat models are highly useful for advancing our understanding of diabetes and its complications, as well as for developing novel treatments for this disease, 20 including the hearing impairment we aim to research.

Curcuminoids, which consist of curcumin, demethoxycurcumin, and bisdemethoxycurcumin, 21 possess anti-inflammatory, antioxidant, and anticancer properties. 22 The United States Food and Drug Administration (FDA) has classified curcumin as safe for human consumption. 23 The therapeutic value of curcuminoids is limited by their instability, low water solubility, and poor bioavailability, particularly at high doses. 24 Curcumin administration at 200 to 400 mg/kg body weight (BW) remains within the safe and tolerable range. Previous studies have demonstrated that a dose of 8,000 mg/day is safe and well tolerated. 25 Additionally, curcuminoid toxicity is not significant. 26 27 Administration of up to 12 grams of curcumin per day has not been associated with adverse effects. 28 Oral curcumin at doses up to 400 mg/kg BW has been found to reduce extracellular matrix production and angiogenic factors in hepatic stellate cells. 29 Furthermore, a 500 mg dose of curcuminoids has been utilized as a therapeutic compound for treating hearing impairment in individuals with chronic kidney disease. 30 Curcumin has also demonstrated safety and efficacy in preventing and mitigating fibroblast damage in the cochlea via cell death pathways. 31 Therefore, we aim to analyze the effects of curcuminoid administration in preventing or repairing damage to the cochlear organ of Corti by examining differences in the mean signal-to-noise ratio (SNR) values, as well as plasma levels of HIF-1α and VEGF, in *Rattus norvegicus* diabetic model rats.

The present study is expected to explore the potential of curcuminoids in preventing or mitigating cochlear damage caused by DM by assessing their effects on SNR, HIF-1α, and VEGF. Additionally, it aims to deepen the understanding of the molecular mechanisms by which curcuminoids regulate inflammatory pathways that support auditory function. The present research has the potential to contribute to the development of curcuminoid-based therapies for DM-related hearing impairment and to advance more effective and safer treatment strategies.

## Methods

### Chemicals

The chemicals used in the present study include: Alloxan (alloxan monohydrate, PubChem ID: 57653881, Sigma-Aldrich), Rat HIF-1α enzyme-linked immunosorbent assay (ELISA) kit (Elabscience), Rat VEGF-A ELISA kit (Sigma-Aldrich), and the anesthetic ketamine (Hameln Pharmaceuticals GmbH).

### Animals

The present study utilized male Wistar rats ( *Rattus norvegicus* ), aged 3 months old (adults), with weights ranging from 200 to 250 grams. The animals were housed in a standardized laboratory equipped with complete facilities and experienced personnel. The maintenance and DPOAE testing of the rats were conducted at the Animal Laboratory, Faculty of Mathematics and Natural Sciences, Universitas Sumatera Utara, Medan, Indonesia. The rats were kept in a setting with a temperature of 22 ± 1 °C and controlled humidity. The lighting system was adjusted to a 12-hour light-dark cycle. Throughout the study, all groups had ad libitum access to identical food and water. The diet was specifically formulated to meet the basic nutritional needs of experimental rats and was consistently applied across all groups. This approach ensured that any observed differences between groups were solely due to the experimental treatment rather than to variations in food or water intake.

In the present study, all experimental animals were ensured to be free from disease and physical injury before use. The health of the rats was routinely monitored throughout the experiment through daily physical examinations, including observations of clinical signs such as appetite, dehydration, activity levels, weight loss, and mortality. Each animal was also examined to confirm the absence of physical injuries or conditions that could affect the experimental outcomes. If any signs of health abnormalities were detected, appropriate actions, such as the removal of the animal from the experiment, were taken in accordance with ethical guidelines. This strict health monitoring was implemented to ensure that the study results were not influenced by uncontrolled health factors.

### Induction of Diabetes

After fasting, diabetes was induced with a single intraperitoneal injection of 150 mg/kg BW alloxan monohydrate. 32 Alloxan was stored at 2 to 8 °C. Injection was completed within 30 seconds. Blood samples were collected from the tail of the rats to measure glycemia. Rats with blood glucose levels > 200 mg/dL within 48 hours after induction were confirmed to have diabetes. 33

### Body Weight and Glycemia Investigations

The body weight and blood glucose levels of the rats were evaluated each morning. Blood samples were obtained from the tail, and glucose levels were assessed utilizing an Autocheck glucometer. (MDSS GmbH).

### Experimental Protocol

Daily examinations were conducted to assess clinical signs such as weight loss, dehydration, and toxicity. The rats were assigned randomly to five groups, with each group containing five rats. The rats were housed in cages according to their treatment group. After allowing the rats to acclimate to the laboratory environment for 14 days, treatments were administered as follows:

Group 1 (G1, *n* = 5): The rats received a single alloxan injection at 150 mg/kg BW. Distortion product otoacoustic emission assessments to evaluate SNR were performed before alloxan induction, on the 1st day of diabetes (when diabetes was confirmed), and subsequently on the 4th, 7th, 10th, and 13th days of DM. This group did not receive curcuminoid.
Group 2 (G2, *n* = 5): The rats received a single alloxan injection at 150 mg/kg BW and were given curcuminoid at 200 mg/kg BW/day. Curcuminoid administration began 72 hours after the diagnosis of diabetes (treatment group 1). Distortion product otoacoustic emission assessments were conducted before alloxan induction, on the 1st day of diabetes, and on the 4th, 7th, 10th, and 13th days of DM.
Group 3 (G3, *n* = 5): The rats received a single alloxan injection at 150 mg/kg BW and were given curcuminoid at 400 mg/kg BW/day. Curcuminoid administration began 72 hours after the diagnosis of diabetes (treatment group 2). Distortion product otoacoustic emission assessments were conducted before alloxan induction, on the 1st day of diabetes, and on the 4th, 7th, 10th, and 13th days of DM.
Group 4 (G4, *n* = 5): The rats received a single alloxan injection at 150 mg/kg BW and were given curcuminoid at 200 mg/kg BW/day starting from the diagnosis of diabetes (prevention group 1). Distortion product otoacoustic emission assessments were conducted before alloxan induction, on the 1st day of diabetes, and on the 4th, 7th, 10th, and 13th days of DM.
Group 5 (G5, *n* = 5): The rats received a single alloxan injection at 150 mg/kg BW and were given curcuminoid at 400 mg/kg BW/day starting from the diagnosis of diabetes (prevention group 2). Distortion product otoacoustic emission assessments were conducted before alloxan induction, on the 1st day of diabetes, and on the 4th, 7th, 10th, and 13th days of DM.

The curcuminoid (Turmeric-Curcumin) used was curcumin 95% turmeric root extract purchased from turmeric-curcumin.com. The curcumin powder was dissolved in carboxy methyl cellulose (CMC). The CMC solution was prepared by suspending 0.5 grams of CMC in 100 ml of distilled water. After suspension, it was administered directly into the rats' stomachs using an orogastric gavage.

### Sample Preparation and Biochemical Analysis

After anesthesia, all rats were euthanized at the end of the study. Blood was collected from the heart of the rats and subjected to centrifugation at 14 thousand rpm for 10 minutes. The resulting supernatant was promptly transferred into tubes using a micropipette. Hypoxia inducible factor- α levels were measured according to the instructions provided with the ELISA Kit. The ELISA Kit employed a Sandwich-ELISA principle. The ELISA microplate was coated with specific antibodies for rat HIF-1α. Standards or samples were introduced into the wells of the microplate and combined with specific antibodies. Wells containing HIF-1α, biotinylated detection antibodies, and avidin-horseradish peroxidase (HRP)-streptavidin conjugate, initially exhibited a blue coloration. However, upon the addition of the stop solution, they underwent a change in coloration, appearing yellow. Optical density was determined spectrophotometrically at 450 ± 2 nm wavelength. The optical density values were proportional to the HIF-1α levels in the samples. Hypoxia inducible factor- α levels were calculated by comparing the optical density of the sample with a standard curve.

Vascular endothelial growth factor A levels were assessed following the guidelines provided in the ELISA Kit. Standards and plasma samples were added to the wells using a micropipette. Vascular endothelial growth factor A in the plasma was bound by the antibodies present in the wells. After washing, specific biotinylated detection antibodies were added. Following another wash, HRP-streptavidin was added to the wells. After washing again, 3,3',5,5'-Tetramethylbenzidine (TMB) substrate solution was added to the wells, producing a color corresponding to the concentration of VEGF-A in the sample. The stop solution converted the blue color to yellow. Color intensity was assessed with a Multiskan Go Spectrophotometer (Thermo Fisher Scientific Oy) at a wavelength of 450 nm.

### Distortion Product Otoacoustic Emission Procedure

Distortion product otoacoustic emission measurements were conducted using the GSI Corti otoacoustic emissions system (Grason-Stadler). Before testing, an injection of 50 mg/kg BW of ketamine (Hameln Pharmaceuticals GmbH) was administered to all rats intramuscularly. 34 After anesthesia, otoscopy was performed, followed by DPOAE testing according to the treatment groups. Testing was conducted in a quiet room. A suitable probe was inserted into the external ear canal of the rats. The testing involved using two simultaneous pure-tone stimuli with different frequencies and intensities. Measurements were conducted at frequencies from 1.5 kHz to 12 kHz (40 to 70 dB HL). A SNR ≥ 3 dB HL was considered a 'pass'. 35 Measurement results were obtained in the form of Distortion Product (DP)-grams expressed in dB HL.

### Statistical Analysis

The Shapiro-Wilk test was employed to assess the normality of the data. The SNR values between groups were calculated based on the mean SNR for each group. Differences in SNR and VEGF-A levels between groups were analyzed using one-way analysis of variance (ANOVA) and post-hoc Games-Howell tests, while HIF-1α levels were evaluated with one-way ANOVA and subsequent Bonferroni tests. Changes in SNR across different time points were analyzed based on the mean SNR at each time point, including prealloxan induction, on the 1st day of diabetes and on days 4th, 7th, 10th, and 13th of DM. Comparisons of SNR values across time points were analyzed using repeated measures ANOVA with post-hoc pairwise comparisons Bonferroni. The mean SNR used to calculate correlations between SNR and HIF-1α and VEGF-A was the mean SNR on the last day (day 13th of DM). Pearson correlation tests were performed to examine correlations between SNR and HIF-1α and VEGF-A plasma levels. Statistical significance was defined as a *p* -value < 0.05.

## Results

**Fig. 1:**
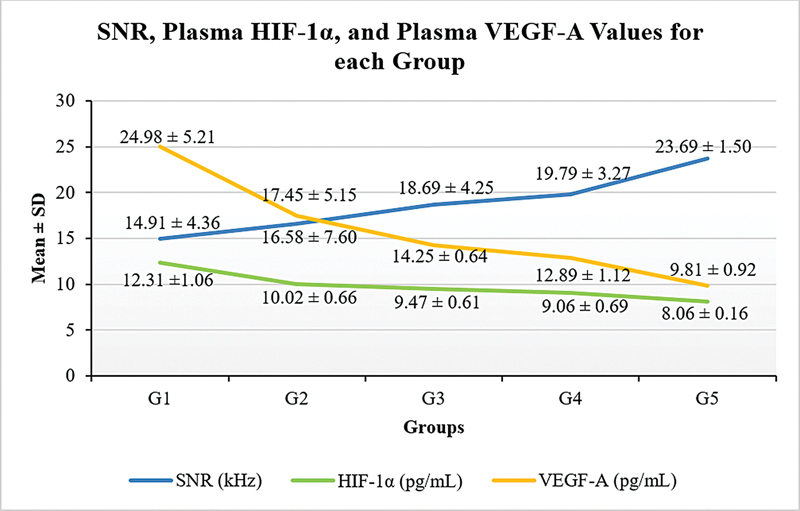
SNR ( *p* = 0.015), plasma HIF-1α ( *p* < 0.001), and plasma VEGF-A ( *p* < 0.001) values for each group; analyzed using ANOVA. Abbreviations: ANOVA, analysis of variance; G, group; HIF-1α, hypoxia-inducible factor-1 alpha; SNR, signal-to-noise ratio; VEGF-A, vascular endothelial growth factor-A.

Figure 1 demonstrates statistically significant differences in the mean SNR values ( *p* = 0.015), plasma HIF-1α levels ( *p* < 0.001), and plasma VEGF-A levels ( *p* < 0.001) among the groups of *Rattus norvegicus* in the DM model following curcuminoid administration. The lowest mean SNR value was observed in the control group that did not receive curcuminoids (G1; 12.31 ± 1.06), whereas the highest value was found in the prevention group receiving a curcuminoid dose of 400 mg/kg BW (G5; 8.06 ± 0.16). Similarly, the most substantial reductions in HIF-1α and VEGF-A levels were also observed in the prevention group administered 400 mg/kg BW of curcuminoids (G5; 8.06 ± 0.16 and 9.81 ± 0.92 respectively). These findings indicate that curcuminoid administration enhances SNR values while simultaneously reducing HIF-1α and VEGF-A levels. To further identify group differences, a post-hoc analysis was conducted ( Table 1 ).

**Table 1:** Statistical significance of the SNR, plasma HIF-1α, and plasma VEGF-A levels among the groups

Variable	Groups		*p* - *value* **
SNR ^a^	G1	G2	0.991
		G3	0.649
		G4	0.348
		G5	0.041*
	G2	G3	0.979
		G4	0.898
		G5	0.375
	G3	G4	0.989
		G5	0.233
	G4	G5	0.234
HIF-1α ^b^	G1	G2	< 0.001*
		G3	< 0.001*
		G4	< 0.001*
		G5	< 0.001*
	G2	G3	1.000
		G4	0.427
		G5	0.002*
	G3	G4	1.000
		G5	0.044*
	G4	G5	0.337
VEGF-A ^a^	G1	G2	0.238
		G3	0.043*
		G4	0.027*
		G5	0.012*
	G2	G3	0.669
		G4	0.418
		G5	0.121
	G3	G4	0.243
		G5	< 0.001*
	G4	G5	0.010*

**Abbreviations:** G, group; HIF-1α, hypoxia-inducible factor-1 alpha; SNR, signal-to-noise ratio; VEGF-A, vascular endothelial growth factor-A.

**Notes:** *Statistically significant ( *p* < 0.05). **Post-hoc ANOVA test. ^a^ Post-hoc Games-Howell. ^b^ Post-hoc Bonferroni.

Table 1 shows that after curcuminoid administration, a statistically significant difference in SNR values was observed between G1 and G5 ( *p* = 0.041). This finding indicates that curcuminoid administration at a preventive dose of 400 mg/kg BW was more effective in improving SNR values compared with the group that did not receive curcuminoids. Table 1 also demonstrates statistically significant differences in plasma HIF-1α levels between G1 and G2, G3, G4, and G5 (each *p* < 0.001). This result suggests that curcuminoid administration led to a statistically significant reduction in plasma HIF-1α levels compared with the untreated group. Additionally, significant differences were found between G2 and G5 ( *p* = 0.002) and between G3 and G5 ( *p* = 0.044), indicating that prevention with a 400 mg/kg BW curcuminoid dose was more effective in reducing plasma HIF-1α levels than treatment with 200 mg/kg BW or 400 mg/kg BW doses. Furthermore, statistically significant differences in plasma VEGF-A levels were observed between G1 and G3 ( *p* = 0.043), G4 ( *p* = 0.027), and G5 ( *p* = 0.012). Significant differences were also found between G3 and G5 ( *p* < 0.001) and between G4 and G5 ( *p* = 0.010), indicating that prevention with a 400 mg/kg BW curcuminoid dose was more effective in reducing plasma VEGF-A levels compared with treatment with a 400 mg/kg BW dose and prevention with a 200 mg/kg BW dose. Therefore, prevention was found to be more effective than treatment.

**Fig. 2:**
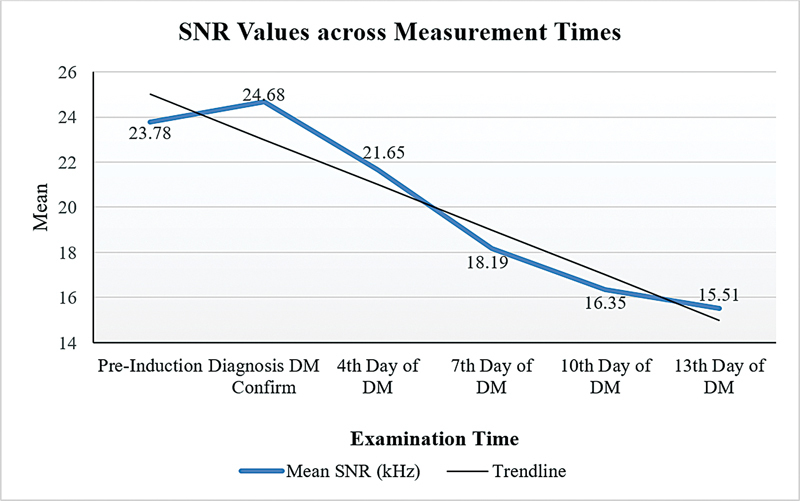
Comparison of SNR values across measurement times; analyzed using repeated measures ANOVA ( *p* < 0.001).

Figure 2 presents statistically significant differences in the mean SNR values across various measurement time points, ranging from prealloxan induction to the 13th day of DM in *Rattus norvegicus* . The results indicate a progressive decline in SNR values as the duration of diabetes increases, reflecting a gradual deterioration of cochlear function over time. Based on the provided data, the mean SNR values show a significant decline over time under diabetic conditions, with an average SNR of 23.78 ± 3.02 recorded during the preinduction measurement and 15.51 ± 6.55 on the 13th day of DM. The decrease in SNR at each measurement point demonstrates the progressive impairment of cochlear function as diabetes progresses. The statistically significant reduction ( *p* < 0.001) further indicates the substantial impact of diabetes on auditory function impairment over time.

Table 2 shows that after the administration of curcuminoids, significant differences in SNR values were identified between the preinduction assessment and the 7th, 10th, and 13th days of DM; SNR values while DM diagnosis confirmed during DM compared to the 4th, 7th, 10th, and 13th days of DM; and SNR values on the 4th day of DM compared to the 7th, 10th, and 13th days of DM. This indicates that the onset of curcuminoid administration affects improving SNR values. The earlier the onset of curcuminoid administration, the more it can improve SNR values.

**Table 2:** Bonferroni pairwise comparisons of the SNR values regarding examination times

Examination time	Versus examination time	Mean difference	95%CI		*p* - *value* **
			Lower bound	Upper bound	
Preinduction	Confirmed DM diagnosis	- 0.89	- 3.53	1.75	1.000
	4th day of DM	2.14	- 0.22	4.49	0.103
	7th day of DM	5.59	2.01	9.18	0.001*
	10th day of DM	7.43	3.51	11.35	< 0.001*
	13th day of DM	8.28	3.50	13.05	< 0.001*
Confirmed DM diagnosis	4th day of DM	3.03	0.52	5.54	0.009*
	7th day of DM	6.49	2.76	10.21	< 0.001*
	10th day of DM	8.32	5.04	11.60	< 0.001*
	13th day of DM	9.17	4.65	13.69	< 0.001*
4th day of DM	7th day of DM	3.46	0.50	6.41	0.013*
	10th day of DM	5.29	2.50	8.08	< 0.001*
	13th day of DM	6.14	2.17	10.10	0.001*
7th day of DM	10th day of DM	1.84	- 1.18	4.86	0.888
	13th day of DM	2.68	-0.89	6.26	0.333
10th day of DM	13th day of DM	0.85	- 2.34	4.04	1.000

**Abbreviations:** DM, diabetes mellitus; SNR, signal-to-noise ratio.

**Notes:** *Statistically significant ( *p* < 0.05). ** Bonferroni pairwise comparisons test.

**Fig. 3:**
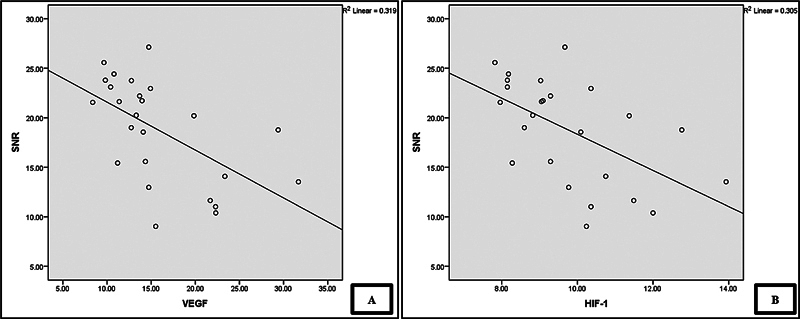
Correlation between SNR values and plasma HIF-1α (A, *p* = 0.004) and VEGF-A (B, *p* = 0.003); analyzed using the Pearson correlation test.

Figure 3 shows a statistically significant correlation between SNR values and plasma levels of HIF-1α and VEGF. The Pearson correlation coefficient is -0.553 for plasma HIF-1α and -0.564 for plasma VEGF, indicating strong and moderate correlation strengths, respectively. Lower levels of plasma HIF-1α and VEGF are associated with higher SNR values.

## Discussion

We found that curcuminoids affect preventing and treating damage to the outer hair cell in the organ of Corti due to DM complications, as evidenced by the improvement in SNR values ( Fig. 1 and Table 1 ) and the correlation between SNR affect values and HIF-1α and VEGF-A ( Fig. 3 ) in groups receiving curcuminoids. The highest plasma levels of HIF-1α and VEGF-A were observed in the control group that did not receive curcuminoids. In contrast, the lowest HIF-1α and VEGF-A plasma levels were found in the 400 mg/kg BW prevention group ( Fig. 1 ). This is in line with other research on the potential effects of curcumin in reducing HIF-1 and VEGF levels in cancer patients under hypoxic conditions. Curcumin can inhibit hypoxia-induced angiogenesis in vascular endothelial cells 36 and improve SNR values in *Rattus norvegicus* exposed to noise. 37 However, this contrasts with previous studies that showed no statistically significant difference in the effect of curcumin on hypoxia-induced angiogenesis. The study on liver tumor cases using liposomal curcumin at a dose of 20 mg/kg BW reported such results. 38 No significant effect was observed in inhibiting the reduction of DPOAE and transient evoked otoacoustic emissions (TEOAE) amplitudes in a DM model exposed to noise. 39 The differences in study results may be attributed to variations in the experimental model, dosage, and administration protocol. Overall, plasma levels of HIF-1α and VEGF-A were significantly different between the control group and the treatment or prevention groups receiving curcuminoids, except for VEGF-A levels in group 2 ( Table 1 ). The effect is dose-dependent and influenced by the duration of administration. 40 We found that curcuminoids administered at a dose of 400 mg/kg BW had better preventive and therapeutic effects compared with a dose of 200 mg/kg BW. Higher doses and longer administration of curcuminoids were associated with lower levels of HIF-1α and VEGF-A. The reduction in HIF-1α and VEGF-A levels also showed similar results ( Fig. 1 ).

The lowest SNR values were found in the control group that did not receive curcuminoids ( Fig. 1 ). The SNR values decreased with the duration of DM ( Fig. 2 ). The decrease in SNR values indicates hearing impairment. 41 We suggest that this decline in SNR is most likely caused by oxidative stress, hypoxia, and cochlear microvascular dysfunction due to chronic hyperglycemia. Diabetes-induced hypoxia increases HIF-1α expression, which stimulates VEGF-A, leading to increased vascular permeability and angiogenesis in the cochlea. This process damages cochlear hair cells, which play a crucial role in sound transduction, ultimately impairing auditory function. 13 14 16 17 Curcuminoids have the potential to protect the cochlea through their antioxidant and anti-inflammatory properties. 42 These compounds suppress HIF-1α expression, mitigate hypoxia-related effects, and inhibit VEGF-A activation. 43 Additionally, HIF plays a role in reducing Reactive Oxygen Species (ROS) production during chronic hypoxia to protect cells while activating the transcription of genes involved in angiogenesis ( *VEGF* ), cell proliferation, and pH regulation. 44 45 Thus, curcuminoids help maintain cochlear microvascular homeostasis and reduce oxidative stress, slowing the progression of hearing impairment in DM. However, the negative effects of DM appear to outweigh the benefits of curcuminoids in preventing or reversing SNR decline.

The best SNR values were also observed in the prevention group receiving a dose of 400 mg/kg BW ( Table 1 ). Administration of curcuminoids in diabetic rats showed that low average DPOAE amplitudes could be improved after treatment with the appropriate dose. 46 Curcuminoids have anti-inflammatory, antioxidant, and antiapoptotic effects. 47 We argue that the ability to prevent cochlear outer hair cell damage is attributed to these effects. Other studies have demonstrated that antioxidant administration represents an efficacious strategy for improving noise-induced hearing impairment in the cochlea. 48

The greatest reduction in HIF-1α levels occurred in the prevention group receiving 400 mg/kg BW ( Fig. 1 ). Statistical analysis confirmed that this prevention dose was more effective than the same treatment dose in reducing HIF-1α levels ( Fig. 1 and Table 1 ). Thus, curcuminoids are more effective in preventing DM-related hearing impairment than in treating it, likely due to their cytoprotective effects against inflammation, lipid peroxidation, and oxidative stress. Curcuminoids inhibit cytochrome activation, preventing ROS accumulation. 43 Curcumin reduces oxidative stress in the blood, improving vascular inflammation in diabetes. 49 It also provides protective effects by suppressing hypoxia-induced gene expression. 50 Under hypoxic conditions, cells regulate inflammation through anti- and proinflammatory mediators, including peptides, glycoproteins, and transcription factors. 51 Hypoxia inducible factor- α , a key regulator of oxygen homeostasis, is activated in response to hypoxia. It controls genes involved in angiogenesis ( *VEGF* ), vasomotor regulation (nitric oxide synthase [NOS]), red blood cell formation, iron metabolism, cell proliferation (insulin-like growth factor-1), and energy metabolism (glucose transporter [GLUT 1–3], phosphofructokinase-1). Given its regulation of essential genes, HIF-1 plays a crucial role, particularly under hypoxic conditions. 52

The effect of curcuminoid administration in reducing VEGF levels was demonstrated in the present study ( Fig. 1 ). Other studies have shown that curcumin lowers VEGF levels in diet-induced hepatocellular carcinoma 53 and reduces VEGF levels induced by osteopontin protein and tumor angiogenesis. 54 Curcumin can decrease cellular activity of various growth factors and cytokines, including VEGF. Curcumin is a traditional medicine with anti-inflammatory properties and can mitigate oxidative stress to maintain cellular homeostasis. 38 Oxidative damage in diabetic patients can be reduced with anti-inflammatory agents 55 and antioxidant intake. 56 The protective anti-inflammatory and antioxidant effects of curcumin can effectively control diabetes. 57 Curcumin is beneficial in preventing and treating various inflammatory diseases through the inhibition of lipoxygenase and cyclooxygenase. 58

The most significant decrease in VEGF-A levels was observed in the prevention group receiving 400 mg/kg BW curcuminoid ( Fig. 1 ). Statistical tests also showed that the 400 mg/kg BW prevention dose was more impactful than the 400 mg/kg BW treatment dose in reducing VEGF-A levels and that the 400 mg/kg BW prevention dose was better than the 200 mg/kg BW prevention dose ( Fig. 1 and Table 1 ). Curcumin is effective in angiogenesis mechanisms depending on the dose and concentration administered. 38 Curcumin can prevent angiogenesis due to its antiangiogenic and antioxidant activities, 59 which effectively lower VEGF levels by preventing angiogenesis responses. 60 Curcumin has high therapeutic efficacy in angiogenesis, tumorigenesis, and signal transduction. Curcumin inhibits VEGF expression, making it useful as a treatment for diseases related to angiogenesis, particularly microvascular complications of diabetes. 61 Curcumin can also address insulin resistance due to DM. 62 Therefore, curcumin can prevent diabetes complications. 43

The 400 mg/kg BW dose used in the present study was still well tolerated, as curcuminoids have a safe dose limit of up to 1,000 mg/kg BW and a lethal dose of 50 (LD50) > 5,000 mg/kg BW in *Rattus norvegicus* . 63 Various studies on animals and humans have demonstrated that curcuminoids are very safe to consume. The safety and pharmacological effectiveness of curcumin position it as a promising compound for the treatment and prevention of various human diseases. 64 The potential of curcuminoids in various biological activities involves multiple mechanisms 65 that can provide radical scavenging effects and enhance biological antioxidant defense systems. 56

The correlation analysis results indicate a strong negative correlation between SNR values and plasma levels of HIF-1α (r = - 0.553; *p* = 0.004) and VEGF-A (r = - 0.564; *p* = 0.003) on Figure 3 . Increased levels of HIF-1α and VEGF-A are associated with decreased SNR values, reflecting cochlear dysfunction. In DM, chronic hypoxia caused by metabolic disturbances leads to increased HIF-1α expression, triggering inflammatory responses and oxidative stress in the cochlea. 13 14 Elevated HIF-1α also contributes to VEGF-A dysregulation, which plays a crucial role in angiogenesis and vascular function in the cochlea. 15 This dysregulation increases vascular permeability, reduces blood flow, and disrupts the delivery of nutrients and oxygen to the cochlea, ultimately exacerbating hearing impairment in diabetic patients. 16 17 The negative correlation observed in the present study supports the hypothesis that hypoxia and excessive angiogenesis contribute to cochlear dysfunction in DM. Curcuminoids play a protective role by reducing hypoxia and inhibiting the VEGF-A pathway. These compounds exhibit antioxidant and anti-inflammatory properties, 42 which can suppress HIF-1α expression, reduce VEGF-A stimulation, and prevent cochlear damage caused by hypoxia and vascular alterations. 43 Therefore, the strong negative correlation between SNR and HIF-1α and VEGF-A further supports the protective mechanism of curcuminoids in preserving cochlear function under diabetic conditions.

We acknowledge that the present research has several limitations, such as a small sample size, the absence of a normal control group for comparison, and the lack of histopathological evidence supporting the protective mechanism of curcuminoids in the cochlea and pancreas. Histopathological analysis is crucial to confirm whether structural improvements align with functional hearing recovery and reduced hypoxia. Furthermore, future research should investigate the long-term effects of curcuminoid administration to determine the sustainability of its therapeutic benefits and side effects. Exploring the combination of curcuminoids with other therapies may also help evaluate potential synergistic effects. Additionally, clinical trials on human subjects are essential to validate these findings in a broader clinical context. With a more comprehensive approach, future research can provide stronger evidence regarding the effectiveness of curcuminoids as a therapeutic strategy for preventing and treating diabetes-related complications.

## Conclusion

In conclusion, the present study confirms that DM causes damage to the outer hair cells of the cochlea, as evidenced by decreased SNR values and increased plasma biomarkers of HIF-1α and VEGF-A. The present study also demonstrates that curcuminoids can prevent and repair damage to the organ of Corti in diabetic *Rattus norvegicus* model, as shown by improvements in SNR values and the correlation between SNR values and plasma levels of HIF-1α and VEGF-A. Curcuminoids also reduce HIF-1α and VEGF-A plasma levels in diabetic *Rattus norvegicus* models. Curcuminoids are a potential therapeutic agent for the prevention and treatment of sensorineural hearing loss caused by DM complications.

## References

[1] International Diabetes Federation IDF Diabetes Atlas 10th edition. Brussels International Diabetes Federation 2021. Available from:https://www.diabetesatlas.org

[2] Kim M B Zhang Y Chang Y Ryu S Choi Y Kwon M J Diabetes mellitus and the incidence of hearing loss: a cohort study Int J Epidemiol 2017 46 02 717 726 10.1093/ije/dyw243 27818377 PMC6251644

[3] Baiduc R R Helzner E P Epidemiology of Diabetes and Hearing Loss Semin Hear 2019 40 04 281 291 10.1055/s-0039-1697643 31602091 PMC6785338

[4] Horikawa C Kodama S Tanaka S Fujihara K Hirasawa R Yachi Y Diabetes and risk of hearing impairment in adults: a meta-analysis J Clin Endocrinol Metab 2013 98 01 51 58 10.1210/jc.2012-2119 23150692

[5] Vesperini E Di Giacobbe F Passatore M Vesperini G Sorgi C Vespasiani G Audiological Screening in People with Diabetes. First Results Audiology Res 2011 1 01 e8 10.4081/audiores.2011.e8 PMC4627155 26557317

[6] Deng Y Chen S Hu J Diabetes mellitus and hearing loss Mol Med 2023 29 01 141 10.1186/s10020-023-00737-z 37875793 PMC10599066

[7] Tsuda J Sugahara K Hori T Kanagawa E Takaki E Fujimoto M A study of hearing function and histopathologic changes in the cochlea of the type 2 diabetes model Tsumura Suzuki obese diabetes mouse Acta Otolaryngol 2016 136 11 1097 1106 10.1080/00016489.2016.1195012 27308832

[8] Taylor R R Jagger D J Forge A Defining the cellular environment in the organ of Corti following extensive hair cell loss: a basis for future sensory cell replacement in the Cochlea PLoS One 2012 7 01 e30577 10.1371/journal.pone.0030577 22299045 PMC3267727

[9] Huo Z Gu J He T Apelin-13 reduces high glucose-induced mitochondrial dysfunction in cochlear hair cells by inhibiting endoplasmic reticulum stress Exp Ther Med 2024 27 05 226 10.3892/etm.2024.12515 38596659 PMC11002831

[10] Goutman J D Elgoyhen A B Gómez-Casati M E Cochlear hair cells: The sound-sensing machines FEBS Lett 2015 589 22 3354 3361 10.1016/j.febslet.2015.08.030 26335749 PMC4641020

[11] Xing Y Ji Q Li X Ming J Zhang N Zha D Lin Y Asiaticoside protects cochlear hair cells from high glucose-induced oxidative stress via suppressing AGEs/RAGE/NF-κB pathway Biomed Pharmacother 2017 86 531 536 10.1016/j.biopha.2016.12.025 28024288

[12] Erkan S O Tuhanioğlu B Gürgen S G Özdaş T Taştekin B Pelit A Görgülü O The effect of resveratrol on the histologic characteristics of the cochlea in diabetic rats Laryngoscope 2019 129 01 E1 E6 10.1002/lary.27253 30284252

[13] Lin Y Shen J Li D Ming J Liu X Zhang N MiR-34a contributes to diabetes-related cochlear hair cell apoptosis via SIRT1/HIF-1α signaling Gen Comp Endocrinol 2017 246 63 70 10.1016/j.ygcen.2017.02.017 28263817

[14] Zhao M Wang S Zuo A Zhang J Wen W Jiang W HIF-1α/JMJD1A signaling regulates inflammation and oxidative stress following hyperglycemia and hypoxia-induced vascular cell injury Cell Mol Biol Lett 2021 26 01 40 10.1186/s11658-021-00283-8 34479471 PMC8414688

[15] Liu F Xia M Xu A Expression of VEGF, iNOS, and eNOS is increased in cochlea of diabetic rat Acta Otolaryngol 2008 128 11 1178 1186 10.1080/00016480801901774 19241604

[16] Maniaci A Briglia M Allia F Montalbano G Romano G L Zaouali M A The Role of Pericytes in Inner Ear Disorders: A Comprehensive Review Biology (Basel) 2024 13 10 802 10.3390/biology13100802 PMC11504721 39452111

[17] Behl T Kotwani A Exploring the various aspects of the pathological role of vascular endothelial growth factor (VEGF) in diabetic retinopathy Pharmacol Res 2015 99 137 148 10.1016/j.phrs.2015.05.013 26054568

[18] Li Y Liu B Li J Xin L Zhou Q Early detection of hearing impairment in type 2 diabetic patients Acta Otolaryngol 2020 140 02 133 139 10.1080/00016489.2019.1680863 31961256

[19] Holt A G Kühl A Braun R D Altschuler R The rat as a model for studying noise injury and otoprotection J Acoust Soc Am 2019 146 05 3681 10.1121/1.5131344 31795688

[20] Al-Awar A Kupai K Veszelka M Szűcs G Attieh Z Murlasits Z Experimental diabetes mellitus in different animal models J Diabetes Res 2016 2016 9.051426E6 10.1155/2016/9051426 PMC4993915 27595114

[21] Nelson K M Dahlin J L Bisson J Graham J Pauli G F Walters M A The Essential Medicinal Chemistry of Curcumin J Med Chem 2017 60 05 1620 1637 10.1021/acs.jmedchem.6b00975 28074653 PMC5346970

[22] Kasetsuwan N Reinprayoon U Uthaithammarat L Sereemaspun A Sae-Liang N Chaichompoo W Suksamrarn A Anti-inflammatory effect of curcuminoids and their analogs in hyperosmotic human corneal limbus epithelial cells BMC Complement Med Ther 2024 24 01 172 10.1186/s12906-024-04448-8 38654265 PMC11040938

[23] Elbialy N S Aboushoushah S F Alshammari W W Long-term biodistribution and toxicity of curcumin capped iron oxide nanoparticles after single-dose administration in mice Life Sci 2019 230 76 83 10.1016/j.lfs.2019.05.048 31128136

[24] Yeh C C Su Y H Lin Y J Chen P J Shi C S Chen C N Chang H I Evaluation of the protective effects of curcuminoid (curcumin and bisdemethoxycurcumin)-loaded liposomes against bone turnover in a cell-based model of osteoarthritis Drug Des Devel Ther 2015 9 2285 2300 10.2147/DDDT.S78277 PMC4408943 25945040

[25] Sahebkar A Curcuminoids for the management of hypertriglyceridaemia Nat Rev Cardiol 2014 11 02 123 10.1038/nrcardio.2013.140-c1 24395048

[26] Ajala O S Innocent-Ugwu D O Okechukwu P U Dada O H Demethoxylated Curcuminoids As Antidiabetic Complication Drug Leads – in Silico Studies African J Pharm Res Dev 2024 16 01 14 25 10.59493/ajopred/2024.1.2

[27] Kelloff G J Crowell J A Hawk E T Steele V E Lubet R A Boone C W Strategy and planning for chemopreventive drug development: clinical development plans II J Cell Biochem Suppl 1996 26(S26):54 71 10.1002/jcb.240630705 9154168

[28] Malik P Mukherjee T K Structure-function elucidation of antioxidative and prooxidative activities of the polyphenolic compound curcumin Chinese Journal of Biology 2014 2014 1 8 10.1155/2014/396708

[29] Xu X Y Meng X Li S Gan R Y Li Y Li H B Bioactivity, health benefits, and related molecular mechanisms of curcumin: Current progress, challenges, and perspectives Nutrients 2018 10 10 1553 10.3390/nu10101553 30347782 PMC6213156

[30] Doostkam A Iravani K Malekmakan L Gholamabbas G Roozbeh J Soltaniesmaeili A The effectiveness of curcumin as a safe agent on hearing threshold improvement in patients with chronic kidney disease: a double-blind, placebo-controlled trial Sci Rep 2024 14 01 17576 10.1038/s41598-024-68572-8 39079962 PMC11289080

[31] Haryuna T SH Riawan W Nasution A Ma'at S Harahap J Adriztina I Curcumin reduces the noise-exposed cochlear fibroblasts apoptosis Int Arch Otorhinolaryngol 2016 20 04 370 376 10.1055/s-0036-1579742 27746842 PMC5063744

[32] Ighodaro O M Adeosun A M Akinloye O A Alloxan-induced diabetes, a common model for evaluating the glycemic-control potential of therapeutic compounds and plants extracts in experimental studies Medicina (Kaunas) 2017 53 06 365 374 10.1016/j.medici.2018.02.001 29548636

[33] Effiong G S Essien G E Comparative effect of *Nauclea latifolia* leaf fractions on blood glucose and lipid profile parameters of alloxan induced-diabetic rats J Med Plants Res 2017 11 24 387 392 10.5897/jmpr2017.6355

[34] Paksoy M Ayduran E Şanlı A Eken M Aydın S Oktay Z A The protective effects of intratympanic dexamethasone and vitamin E on cisplatin-induced ototoxicity are demonstrated in rats Med Oncol 2011 28 02 615 621 10.1007/s12032-010-9477-4 20300971

[35] Ceylan S M Uysal E Altinay S Sezgin E Bilal N Petekkaya E Protective and therapeutic effects of milrinone on acoustic trauma in rat cochlea Eur Arch Otorhinolaryngol 2019 276 07 1921 1931 10.1007/s00405-019-05417-5 30955065

[36] Zahedi M Salmani Izadi H Arghidash F Gumpricht E Banach M Sahebkar A The effect of curcumin on hypoxia in the tumour microenvironment as a regulatory factor in cancer Arch Med Sci 2023 19 06 1616 1629 10.5114/aoms/171122 38058727 PMC10696979

[37] Haryuna T SH Amellya D Munir D Zubaidah T SH The benefits of curcuminoid to expression nuclear factor erythroid 2 related factor 2 (NRF2) and signal to noise ratio (SNR) Value in the noise exposed organ of Corti of *Rattus norvegicus* Rep Biochem Mol Biol 2021 10 03 373 379 10.52547/rbmb.10.3.373 34981013 PMC8718775

[38] Dai F Zhang X Shen W Chen J Liu L Gao G Liposomal curcumin inhibits hypoxia-induced angiogenesis after transcatheter arterial embolization in VX2 rabbit liver tumors OncoTargets Ther 2015 8 2601 2611 10.2147/OTT.S87931 PMC4592055 26451117

[39] Spankovich C Long G R Hood L J Early indices of reduced cochlear function in young adults with type-1 diabetes revealed by DpOAE fine structure J Am Acad Audiol 2019 30 06 459 471 10.3766/jaaa.17113 30461415

[40] Ströfer M Jelkmann W Depping R Curcumin decreases survival of Hep3B liver and MCF-7 breast cancer cells: the role of HIF Strahlenther Onkol 2011 187 07 393 400 10.1007/s00066-011-2248-0 21713389

[41] Kumar P Singh N K Apeksha K Ghosh V Kumar R R Muthaiah B K Auditory and vestibular functioning in individuals with type-2 diabetes mellitus: A systematic review Int Arch Otorhinolaryngol 2021 26 02 e281 e288 10.1055/s-0041-1726041 35602282 PMC9122769

[42] Sökmen M Akram Khan M The antioxidant activity of some curcuminoids and chalcones Inflammopharmacology 2016 24(2-3):81 86 10.1007/s10787-016-0264-5 27188988 PMC4883448

[43] Bahrami A Atkin S L Majeed M Sahebkar A Effects of curcumin on hypoxia-inducible factor as a new therapeutic target Pharmacol Res 2018 137 159 169 10.1016/j.phrs.2018.10.009 30315965

[44] Catrina S B Impaired hypoxia-inducible factor (HIF) regulation by hyperglycemia J Mol Med (Berl) 2014 92 10 1025 1034 10.1007/s00109-014-1166-x 25027070

[45] Span P N Bussink J Biology of hypoxia Semin Nucl Med 2015 45 02 101 109 10.1053/j.semnuclmed.2014.10.002 25704383

[46] Özdaş S Taştekin B Gürgen S G Özdaş T Pelit A Erkan S O Pterostilbene protects cochlea from ototoxicity in streptozotocin-induced diabetic rats by inhibiting apoptosis PLoS One 2020 15 07 e0228429 10.1371/journal.pone.0228429 32722679 PMC7386625

[47] Fu Y S Chen T H Weng L Huang L Lai D Weng C F Pharmacological properties and underlying mechanisms of curcumin and prospects in medicinal potential Biomed Pharmacother 2021 141 111888 10.1016/j.biopha.2021.111888 34237598

[48] Pisani A Paciello F Montuoro R Rolesi R Galli J Fetoni A R Antioxidant Therapy as an Effective Strategy against Noise-Induced Hearing Loss: From Experimental Models to Clinic Life 2023 13 04 1 16 10.3390/life13041035 PMC10144536 37109564

[49] Hao M Chu Y Lei J Yao Z Wang P Chen Z Pharmacological Mechanisms and Clinical Applications of Curcumin: Update Aging Dis 2023 14 03 716 749 10.14336/AD.2022.1101 37191432 PMC10187702

[50] Chung L C Tsui K H Feng T H Lee S L Chang P L Juang H H Curcumin provides potential protection against the activation of hypoxia and prolyl 4-hydroxylase inhibitors on prostate-specific antigen expression in human prostate carcinoma cells Mol Nutr Food Res 2011 55 11 1666 1676 10.1002/mnfr.201100328 21936051

[51] Biddlestone J Bandarra D Rocha S The role of hypoxia in inflammatory disease (review) Int J Mol Med 2015 35 04 859 869 10.3892/ijmm.2015.2079 25625467 PMC4356629

[52] Sharp F R Bernaudin M HIF1 and oxygen sensing in the brain Nat Rev Neurosci 2004 5 06 437 448 10.1038/nrn1408 15152194

[53] Mohammed E S El-Beih N M El-Hussieny E A El-Ahwany E Hassan M Zoheiry M Effects of free and nanoparticulate curcumin on chemically induced liver carcinoma in an animal model Arch Med Sci 2020 17 01 218 227 10.5114/aoms.2020.93739 33488874 PMC7811328

[54] Chakraborty G Jain S Kale S Raja R Kumar S Mishra R Kundu G C Curcumin suppresses breast tumor angiogenesis by abrogating osteopontin-induced VEGF expression Mol Med Rep 2008 1 05 641 646 10.3892/mmr_00000005 21479462

[55] Kunnumakkara A B Bordoloi D Padmavathi G Monisha J Roy N K Prasad S Aggarwal B B Curcumin, the golden nutraceutical: multitargeting for multiple chronic diseases Br J Pharmacol 2017 174 11 1325 1348 10.1111/bph.13621 27638428 PMC5429333

[56] Panahi Y Khalili N Sahebi E Namazi S Karimian M S Majeed M Sahebkar A Antioxidant effects of curcuminoids in patients with type 2 diabetes mellitus: a randomized controlled trial Inflammopharmacology 2017 25 01 25 31 10.1007/s10787-016-0301-4 27928704

[57] Mahmoudi A Atkin S L Nikiforov N G Sahebkar A Therapeutic Role of Curcumin in Diabetes: An Analysis Based on Bioinformatic Findings Nutrients 2022 14 15 3244 10.3390/nu14153244 35956419 PMC9370108

[58] Nawaz M I Alhomida A S Ola M S The potential beneficial effects of curcumin in diabetic retinopathy Cambridge, MA Academic Press 2019 401 417 10.1016/B978-0-12-815461-8.00022-0

[59] Sharma A V Ganguly K Paul S Maulik N Swarnakar S Curcumin heals indomethacin-induced gastric ulceration by stimulation of angiogenesis and restitution of collagen fibers via VEGF and MMP-2 mediated signaling Antioxid Redox Signal 2012 16 04 351 362 10.1089/ars.2011.4232 21942294

[60] Wang T Y Chen J X Effects of curcumin on vessel formation insight into the pro- and antiangiogenesis of curcumin Evid Based Complement Alternat Med 2019 2019 1.390795E6 10.1155/2019/1390795 PMC6607718 31320911

[61] Mrudula T Suryanarayana P Srinivas P NBS Reddy G B Effect of curcumin on hyperglycemia-induced vascular endothelial growth factor expression in streptozotocin-induced diabetic rat retina Biochem Biophys Res Commun 2007 361 02 528 532 10.1016/j.bbrc.2007.07.059 17662242

[62] Rivera-Mancía S Trujillo J Chaverri J P Utility of curcumin for the treatment of diabetes mellitus: Evidence from preclinical and clinical studies J Nutr Intermed Metab 2018 14 29 41 10.1016/j.jnim.2018.05.001

[63] Ibrahim J Kabiru A Y Abdulrasheed-Adeleke T Lawal B Adewuyi A H Antioxidant and hepatoprotective potentials of curcuminoid isolates from turmeric ( *Curcuma longa* ) rhizome on CCl _4_ -induced hepatic damage in Wistar rats J Taibah Univ Sci 2020 14 01 908 915 10.1080/16583655.2020.1790928

[64] Anand P Kunnumakkara A B Newman R A Aggarwal B B Bioavailability of curcumin: problems and promises Mol Pharm 2007 4 06 807 818 10.1021/mp700113r 17999464

[65] Amalraj A Pius A Gopi S Gopi S Biological activities of curcuminoids, other biomolecules from turmeric and their derivatives - A review J Tradit Complement Med 2016 7 02 205 233 10.1016/j.jtcme.2016.05.005 28417091 PMC5388087

